# An unexpected tautomer: synthesis and crystal structure of *N*-[6-amino-4-(methyl­sulfan­yl)-1,2-di­hydro-1,3,5-triazin-2-yl­idene]benzenesulfonamide

**DOI:** 10.1107/S2056989023011076

**Published:** 2024-01-09

**Authors:** Reham A. Mohamed-Ezzat, Galal H. Elgemeie, Peter G. Jones

**Affiliations:** a Chemistry of Natural and Microbial Products Department, Pharmaceutical and Drug Industries Research Institute, National Research Centre, Cairo, Egypt; b Chemistry Department, Faculty of Science, Helwan University, Cairo, Egypt; c Institut für Anorganische und Analytische Chemie, Technische Universität Braunschweig, Hagenring 30, D-38106 Braunschweig, Germany; Universität Greifswald, Germany

**Keywords:** crystal structure, 1,3,5-triazine, benzene­sulfonamide, hydrogen bonds

## Abstract

The title compound, C_10_H_11_N_5_O_2_S_2_, consists of an unexpected tautomer with a protonated nitro­gen atom in the triazine ring and a formal exocyclic double bond C=N to the sulfonamide moiety. The ring angles at the unsubstituted nitro­gen atoms are narrow, at 115.57 (12) and 115.19 (12)°, respectively, whereas the angle at the carbon atom between these N atoms is very wide, 127.97 (13)°. The inter­planar angle between the two rings is 79.56 (5)°. The mol­ecules are linked by three classical hydrogen bonds, forming a ribbon structure. There are also unusual linkages involving three short contacts (< 3 Å) from a sulfonamide oxygen atom to the C—NH—C part of a triazine ring.

## Abstract 2

The title compound, C_10_H_11_N_5_O_2_S_2_, consists of an unexpected imino-di­hydro-triazine tautomer. Mol­ecules are linked by hydrogen bonds and by O_sulf­on­amide_⋯(C—NH—C)_triazine_ contacts.

## 1. Chemical context

**Figure 1:**
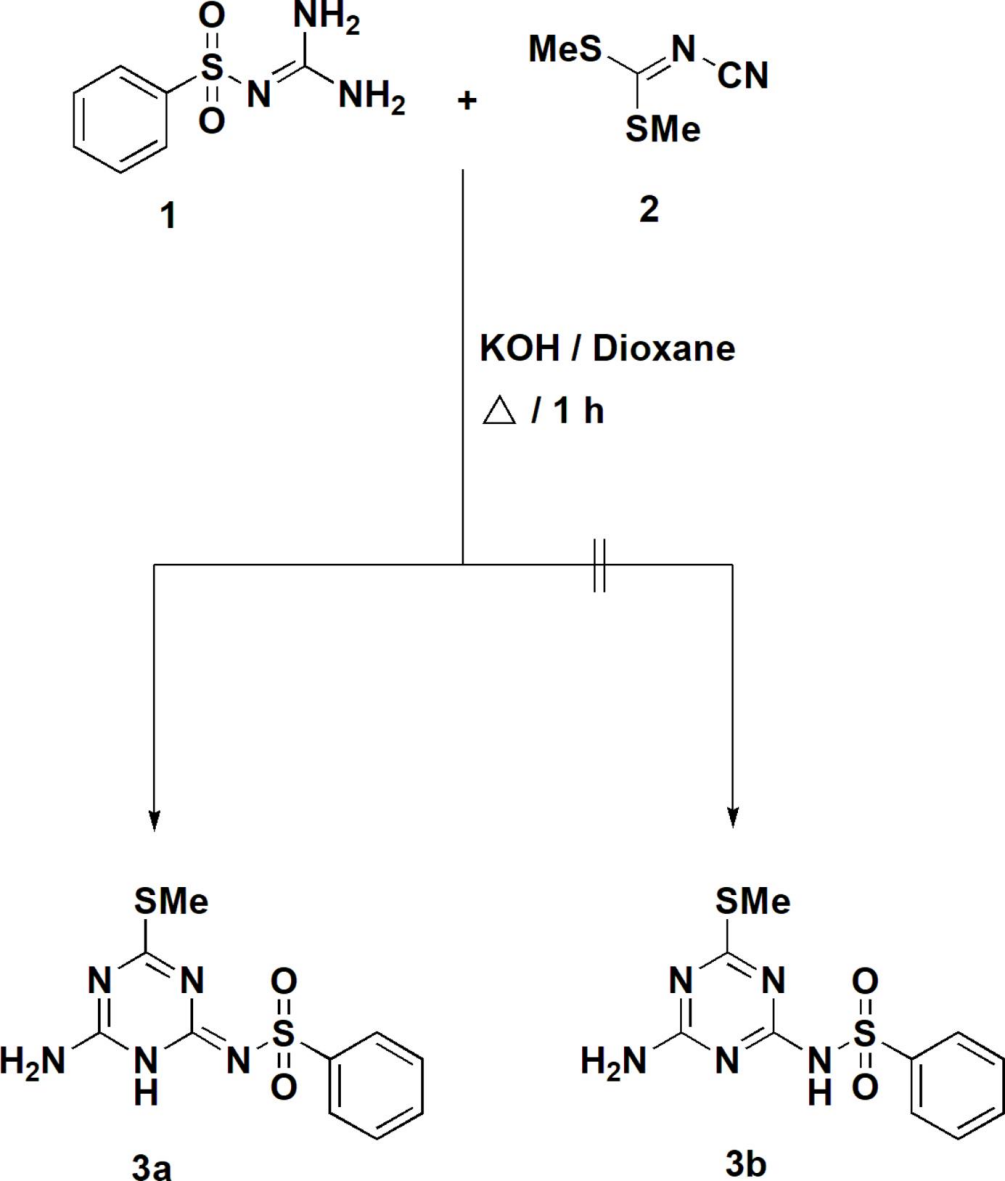
Synthesis of the novel triazine sulfonamide derivative **3a**.

Sulfonamides constitute a significant category of bioactive mol­ecules with remarkable pharmacological activities (Wan *et al.*, 2021 ▸; Elgemeie *et al.*, 2022 ▸). They are clinically utilized as anti­cancer (Owa & Nagasu, 2000 ▸), anti­bacterial, anti­thyroid, hypoglycaemic and anti­viral drugs; among many other effective mol­ecules one may cite the anti-cancer agent indisulam (Supuran, 2003 ▸). The presence of a moiety with a triazine core (as an aza-pyrimidine analogue) would represent a new structure of significant importance. Continuing with our project of developing synthetic strategies for the design and synthesis of efficient anti­metabolites (Elgemeie & Mohamed-Ezzat, 2022 ▸), focussing on derivatives of sulfonamides, we describe here a new approach (Fig. 1 ▸) that generates novel substituted triazine sulfonamides starting from the highly reactive compound dimethyl cyano­carboimidodi­thio­ate (**2**), which has shown its effectiveness in synthesizing various heterocycles (Elgemeie & Mohamed, 2014 ▸ *;* Mohamed-Ezzat *et al.*, 2021 ▸), in particular nucleoside and non-nucleoside pyrimidine analogues (Elgemeie *et al.*, 2015 ▸, 2017 ▸, 2019 ▸).

Thus, the reaction of benzene­sulfonyl­guanidine **1** with the *N*-cyano­dithio­imino­carbonate derivative **2** in refluxing dioxane containing potassium hydroxide for 1 h provided an adduct for which two possible tautomeric structures **3a** or **3b** (derivatives of 1,3,5-triazine, also known as *s*-triazine, with a benzene­sulfonamide substituent) might be assigned (Fig. 1 ▸). Investigation by TLC and NMR revealed the presence of only one product in solution. The ^1^H NMR spectrum of the product showed three singlet signals at δ = 2.29, 7.35 and 11.83 ppm, assigned to SCH_3_, NH_2_ and NH protons, in addition to signals from the aromatic protons; it is, however, inconclusive in differentiating between the two tautomers. An X-ray structure determination, described in this paper, indicated unambiguously the formation of the di­hydro-1,3,5-triazine-benzene­sulfonamide derivative, the title compound **3a**, as the isolated product in the solid state. This compound consists of two important substructures (the sulfonamide and the triazine moieties) and this may prove to have a significant impact in developing the medicinal chemistry of sulfonamides. 
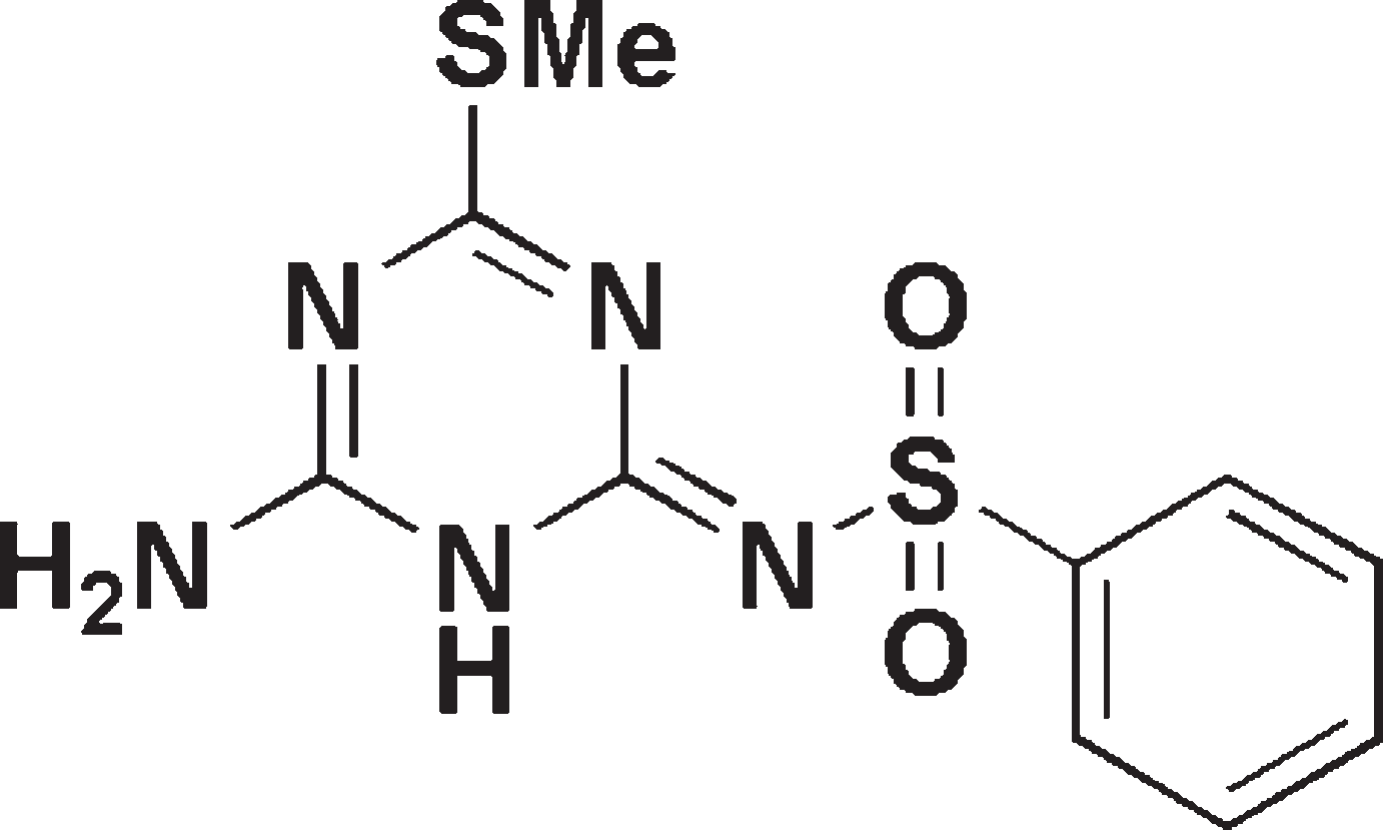


## 2. Structural commentary

**Figure 2:**
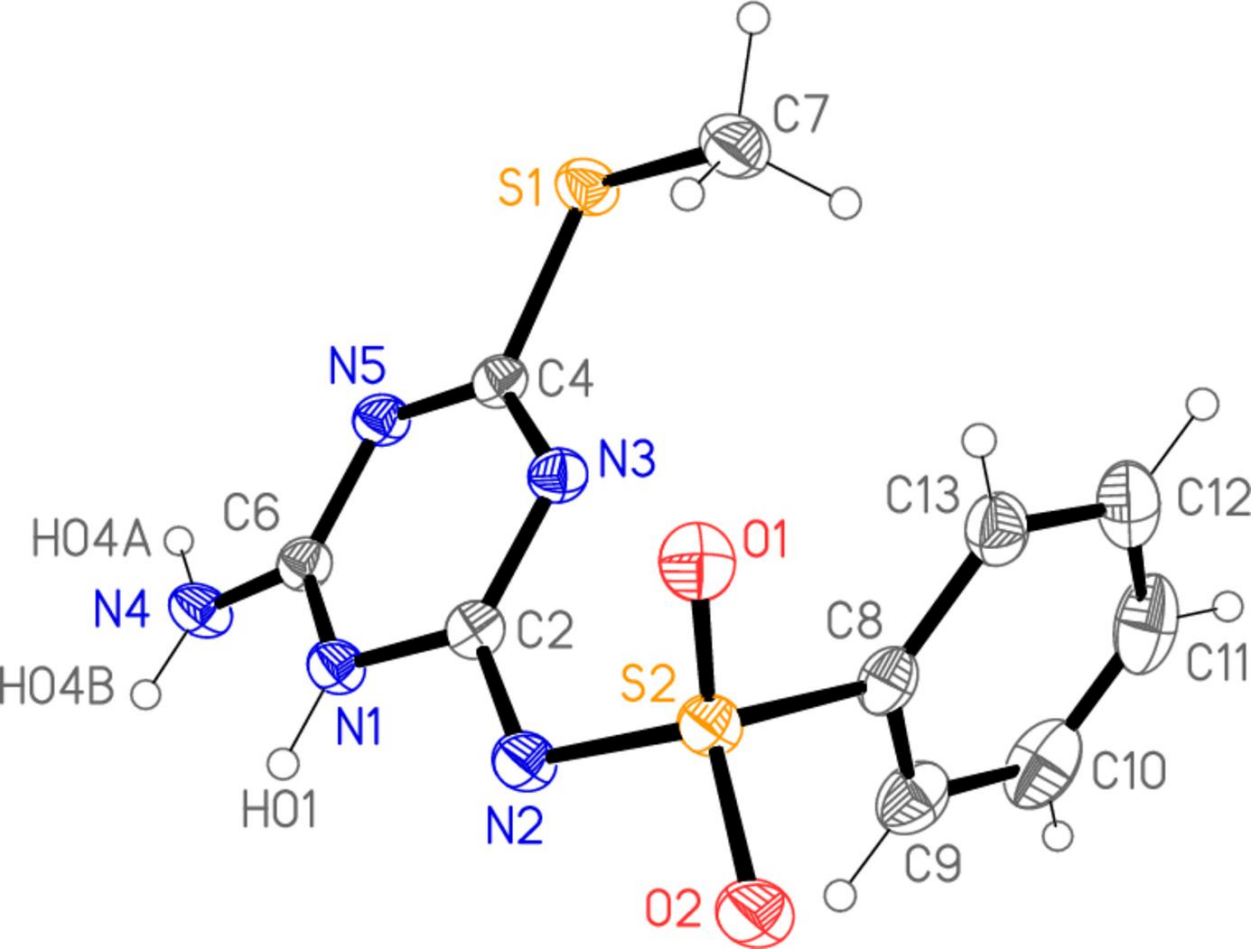
The mol­ecule of **3a** in the crystal. Ellipsoids represent 50% probability levels.

The structure of **3a** is shown in Fig. 2 ▸, with selected mol­ecular dimensions in Table 1 ▸. Surprisingly, the alternative tautomer **3b**, *N*-[6-amino-4-(methyl­sulfan­yl)-1,3,5-triazin-2-yl]benzene­sulfonamide, in which the hydrogen atom at N1 is shifted to N2 (using the numbering of Fig. 2 ▸) was not formed, at least not in significant amounts. It should be stressed that the three hydrogen atoms bonded to nitro­gen were identified in a difference synthesis and refined freely.

The inter­planar angle between the two rings is 79.56 (5)°; the phenyl ring, which is almost ideally planar (r.m.s. deviation = 0.0015 Å), is oriented such that C13 is approximately synperiplanar to O1, with an O1—S2—C8—C13 torsion angle of 7.00 (14)° and a short intra­molecular contact O1⋯H13 2.48 Å. The modified triazine ring, with formal single bonds at N1 and a formal exocyclic double bond C2=N2, has a higher r.m.s. deviation from planarity of 0.03 Å. This is associated with a significant deviation at the nitro­gen atom N1, which lies 0.112 (2) Å out of the plane of the other five atoms, although it retains its planarity (angle sum of 359.3°). Accordingly, the ring torsion angles involving N1 differ appreciably from zero, at *ca* ±10°. The substituents at the triazine ring are also somewhat displaced from the ring plane, N2 by 0.171 (2), N4 by 0.141 (2) and S1 by −0.134 (2) Å. The NH_2_ group is essentially planar (angle sum of 358.2°) and almost coplanar with the triazine ring (its hydrogen atoms lie less than 0.1 Å out of the ring plane).

The modified *s*-triazine ring departs considerably from the threefold local symmetry of unmodified *s*-triazine. The bond lengths are approximately equal [range 1.327–1.373 Å, with the formal single bonds at N1 being the longest], but the angles are markedly different; whereas the angles at C2, N1 and C6 are within 1° of 120°, those at N3 and N5 are appreciably narrower at 115.57 (12) and 115.19 (12)° respectively, and N3—C4—N5 is very wide at 127.97 (13)°. These latter values are reminiscent of the structure of the parent ring system *s*-triazine, which was determined by Wheatley (1955 ▸), with impressive precision for that time; the ring was shown to have crystallographic threefold symmetry, with angles of 126.8 (4)° at carbon and of 113.2 (4)° at nitro­gen. Later investigations by Coppens (1967 ▸) gave values of 126.6° and 113.4° (no e.s.d.’s quoted). Furthermore, the formally double C—N bonds of the ring, C6—N5 [1.3398 (19) Å] and C4—N3 [1.3270 (18) Å], and the exocyclic bond C2—N2 [1.3326 (18) Å], are actually longer than the formal single bond C6—N4 of 1.3144 (19) Å. This shows that the single ‘resonance’ form shown for **3a** is, unsurprisingly, too simple, and that other forms with three formal double bonds in the ring and a single bond for S2—N2 [bond length = 1.6279 (13) Å] should be considered. This view is supported by the value of 1.633 Å for the ‘standard’ N—S bond length in the moiety C—SO_2_—NH—C (Allen *et al.*, 1987 ▸).

## 3. Supra­molecular features

**Figure 3:**
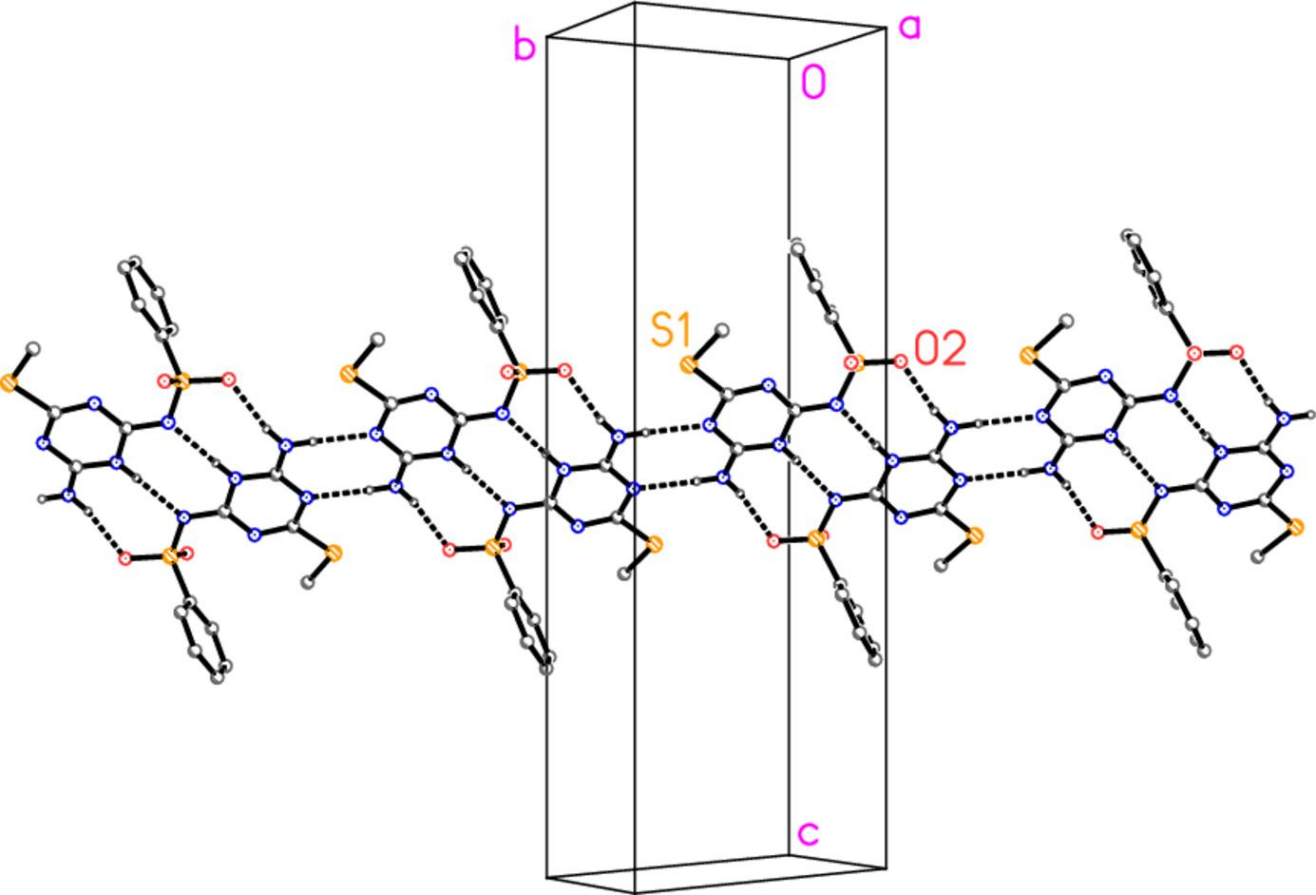
Packing diagram of **3a**, viewed perpendicular to (110), showing the formation of a ribbon of mol­ecules linked by classical hydrogen bonds (dashed lines). Labelled atoms indicate the asymmetric unit. Hydrogen atoms not involved in hydrogen bonding are omitted.

The mol­ecules of **3a** are linked by a series of classical hydrogen bonds (Table 2 ▸), forming a ribbon structure (Fig. 3 ▸). One set of such ribbons, in the region *z* ≃ 0.5, is parallel to [1 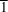 0]; further sets at *z* ≃ 0 and 1 are parallel to [110]. All three potential donor hydrogen atoms (H01, H04*A*, H04*B*) are involved; the respective acceptors are the exocyclic nitro­gen atom N2, the ring nitro­gen atom N5, and the sulfonyl oxygen O2. The hydrogen-bonded rings all have graph set 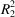 (8) (Bernstein *et al.*, 1995 ▸). In the fused set of three rings, formed *via* the inversion operator 1 − *x*, −*y*, 1 − *z*, the outer rings are anti­dromic whereas the central ring is homodromic. The single ring based on the H04*A*⋯N5 inter­action is also formed by inversion (−*x*, 1 − *y*, 1 − *z*) and is homodromic.

**Figure 4:**
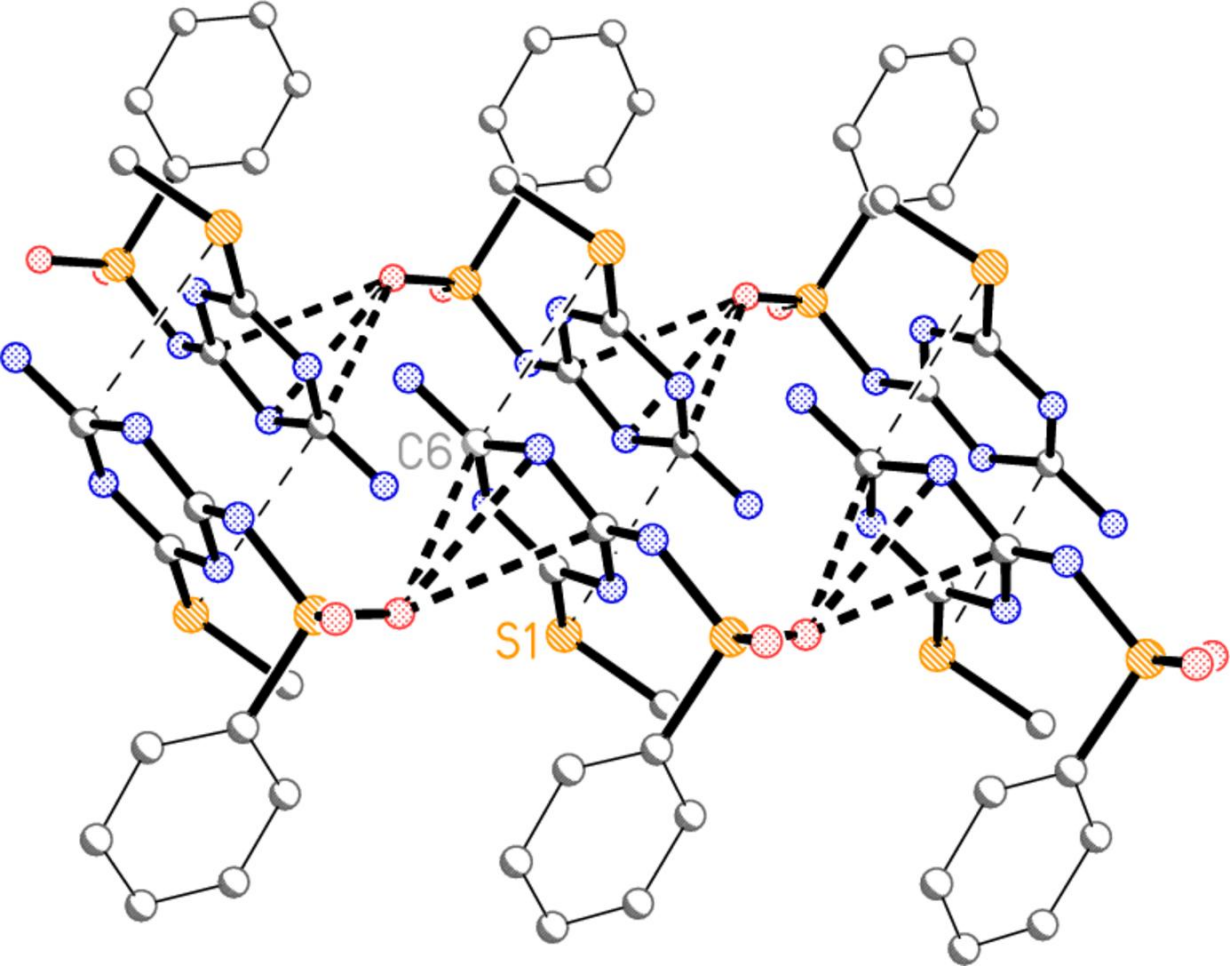
Formation of chains of mol­ecules **3a** parallel to the *a* axis, showing the short contacts between O1 and the triazine ring of a neighbouring mol­ecule (thick dashed bonds). The view direction is approximately parallel to the *b* axis, and the *a* axis runs horizontally. Two such chains, running mutually anti­parallel, are connected by the borderline contacts S1⋯C6 (thin dashed lines). Hydrogen atoms are omitted.

There are no short H⋯centroid or centroid⋯centroid contacts. However, the sulfonyl oxygen atom O1 makes short contacts to three atoms of the triazine ring of a neighbouring mol­ecule related by translation (operator 1 + *x*, *y*, *z*), namely O1⋯C2 = 2.9684 (17), O1⋯N1 = 2.9119 (16) and O1⋯C6 = 2.8883 (18) Å. The resulting chains of mol­ecules are shown in Fig. 4 ▸, in which the borderline contact S1⋯C6 = 3.4846 (15) Å (operator 1 − *x*, 1 − *y*, 1 − *z*) is also included.

## 4. Database survey

The search employed the routine ConQuest (Bruno *et al.*, 2002 ▸), part of Version 2022.3.0 of the CSD (Groom *et al.*, 2016 ▸).

Some adducts of unsubstituted *s*-triazine have been determined and confirm its usual geometry, with angles at nitro­gen of around 114° and at carbon of around 126°: the 1:1 1,2,3-tri­hydroxy­benzene adduct (JAXSOR; Dobrzańska, 2005 ▸); the 1:2 *N*-iodo­succinimide adduct, involving very short N⋯I contacts (IBIZEA; Raatikainen & Rissanen, 2011 ▸) and the 1:1 adduct with thio­cyanuric acid (FOSDUP; Argent *et al.*, 2019 ▸).

We wished to determine how unusual the protonation at the *s*-triazine ring was, in comparison to protonation at an N-substituent of this ring. Accordingly, the following searches were carried out: (i) *s*-triazine ring framework; ‘organic’ structures only; substituent —NH—AA at one carbon atom (AA = any ‘acyclic’ atom), AA at the other C atoms; any bond order for the ring and the exocyclic N—AA; three binding partners for the carbon atoms and the exocyclic nitro­gen, two for the ring nitro­gen atoms. This gave 345 hits; restraining the search to the substituent —NH—S reduced this to just four hits, two involving benzene­sulfonamide derivatives of diethyl-*s*-triazine (LOCHUH and LOCJAP; Haddow *et al.*, 2008 ▸) and two with bis-alkanesulfinimide derivatives of phenyl- (PIMHOL; Zuo *et al.*, 2018*b* ▸) or thio­phen-2-yl-*s*-triazine (QOCCET; Zuo *et al.*, 2018*a* ▸). (ii): as for (i) but with one ring nitro­gen atom protonated and with three binding partners in total, and the exocyclic nitro­gen unprotonated and with two binding partners. This gave only five hits for any N—AA and no hits for N—S, in both cases with unrestricted bond order at this nitro­gen. Four of the hits involved salts of the monoprotonated tri­cyano­melaminate anion (melamine = 1,3,5-triazine-2,4,6-tri­amine) (CEKGUV, CEKHAC, KIFQAS, KIFQEW; Lotsch & Schnick, 2006 ▸, 2007 ▸) and the other, also a melamine derivative, contained a cation with two N=PPh_3_ and one NH_2_ substituent (PUYQUW; Saplinova *et al.*, 2010 ▸). It thus seems that tautomers of *s*-triazine derivatives resembling **3a** may reasonably be described as unusual, especially for uncharged species. The first search however (correctly) failed to find the related zwitterionic species [(6-ethyl­amino)-4-meth­oxy-1,3,5-triazin-1-ium-2-yl](di­nitro)­methanide (YOW­LUS; Bakharev & Gidaspov, 2007 ▸), because this has a protonated ring nitro­gen as well as an NH—AA substituent.

Finally, we searched for short inter­molecular contacts from sulfonyl­amide oxygen atoms to three consecutive atoms of any six-membered ring. There were 49 hits with all contacts shorter than the sum of the CCDC van der Waals radii, but only one structure had all three contacts shorter than 3 Å; a high-pressure study of the drug chloro­thia­zide (6-chloro-4*H*-1,2,4-benzo­thia­diazine-7-sulfonamide 1,1-dioxide, QQQAUG14; Oswald *et al.*, 2010 ▸). Three structures (GEKNAO, GEKNES, PSULTZ) had impossibly short contacts (as low as 2.14 Å), and we suspect serious errors in these structures. The first two (Goyal *et al.*, 2018 ▸) are powder determinations with *R* values of 0.139 and 0.169 respectively, whereas the third (Rivero *et al.*, 1978 ▸) may involve an incorrect space group (as commented in the CCDC entry) or wrongly permuted axes. The structures IGISOH {dimethyl 2,2′-[(3-oxo-3*H*-phenoxazine-1,9-di­yl)bis­(sulfonyl­imino)]di­acetate; Bruyneel *et al.*, 2009 ▸} and HINVOS (1,1′-bis­[4-(dec­yloxy)phen­yl]-4,4′-bipyridin-1-ium bis­{bis­[(tri­fluoro­meth­yl)sulfon­yl]amide}; Ahumada, 2018 ▸) have contacts in the range 2.87–3.03 Å. The contacts in **3a** may thus be described as unusually short but not unprecedented.

## 5. Synthesis and crystallization

A mixture of benzene­sulfonyl­guanidine (**1**) (0.01 mol) and dimethyl cyano­carboimidodi­thio­ate **2** (0.01 mol) in dry dioxane (20 mL) containing potassium hydroxide (0.01 mol) was refluxed for 1 h. The reaction mixture was poured into ice–water and the resulting mixture neutralized with hydro­chloric acid. The precipitate thus formed was filtered off, washed thoroughly with water, dried and crystallized from di­methyl­sulfoxide to obtain compound **3** as pale-yellow crystals in 87% yield. M.p. 520–522 K; IR (KBr, cm^−1^): ν 3261, 3202 (NH), 3065 (Ar—CH), 2931, 2813 (methyl CH), 1555 (C=C), 1358, 1141 (SO_2_); ^1^H NMR (400 MHz, DMSO-*d_6_*): δ 2.29 (*s*, 3H, CH_3_), 7.35 (*s*, 2H, NH_2_), 7.54–7.64 (*m*, 3H, Ar-H), 7.96–7.98 (*d*, 2H, Ar—H), 11.83 (*s*, 1H, NH); ^13^C NMR (400 MHz, DMSO-*d*_6_) δ (ppm): 125.30, 127.75, 128.75, 132.79, 140.85, 159.76, 163.42, 180.34. Analysis calculated for C_10_H_11_N_5_O_2_S_2_ (297.36): C 40.39, H 3.73, N 23.55, S 21.57. Found: C 40.38, H 3.72, N 23.55, S 21.56%.

## 6. Refinement

Crystal data, data collection and structure refinement details are summarized in Table 3 ▸. Hydrogen atoms bonded to nitro­gen were refined freely. The methyl group was included as an idealized rigid group allowed to rotate but not tip (command ‘AFIX 137’). Other hydrogen atoms were included using a riding model starting from calculated positions (C—H = 0.95 Å). The *U*(H) values were fixed at 1.5 × *U*_eq_ of the parent carbon atoms for the methyl group and 1.2 × *U*_eq_ for other hydrogens.

## Supplementary Material

Crystal structure: contains datablock(s) I, global. DOI: 10.1107/S2056989023011076/yz2047sup1.cif

Structure factors: contains datablock(s) I. DOI: 10.1107/S2056989023011076/yz2047Isup2.hkl

Supporting information file. DOI: 10.1107/S2056989023011076/yz2047Isup3.cml

CCDC reference: 2321847

Additional supporting information:  crystallographic information; 3D view; checkCIF report

## Figures and Tables

**Table 1:** Selected geometric parameters (Å, °)

N1—C6	1.3637 (18)	C4—N5	1.3379 (19)
N1—C2	1.3725 (18)	C4—S1	1.7451 (15)
C2—N2	1.3326 (18)	N5—C6	1.3398 (19)
C2—N3	1.3410 (19)	C6—N4	1.3144 (19)
N3—C4	1.3270 (18)	N2—S2	1.6279 (13)
			
C6—N1—C2	119.71 (13)	C4—N5—C6	115.19 (12)
N3—C2—N1	120.15 (13)	N5—C6—N1	120.59 (13)
C4—N3—C2	115.57 (12)	C2—N2—S2	117.22 (10)
N3—C4—N5	127.97 (13)		
			
N3—C2—N2—S2	−0.3 (2)	O1—S2—C8—C13	7.00 (14)
N3—C4—S1—C7	−6.77 (14)		

**Table 2:** Hydrogen-bond geometry (Å, °)

*D*—H⋯*A*	*D*—H	H⋯*A*	*D*⋯*A*	*D*—H⋯*A*
N1—H01⋯N2^i^	0.86 (2)	2.11 (2)	2.9701 (18)	178 (2)
N4—H04*A*⋯N5^ii^	0.87 (2)	2.06 (2)	2.9245 (18)	177 (2)
N4—H04*B*⋯O2^i^	0.83 (2)	1.97 (2)	2.7912 (18)	166 (2)
C7—H7*C*⋯O2^iii^	0.98	2.63	3.445 (2)	140

Symmetry codes: (i) 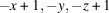 ; (ii) 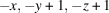 ; (iii) 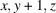 .

**Table 3:** Experimental details

Crystal data	
Chemical formula	C_10_H_11_N_5_O_2_S_2_
*M*_r_	297.36
Crystal system, space group	Monoclinic, *P*2_1_/*n*
Temperature (K)	100
*a*, *b*, *c* (Å)	5.44832 (9), 9.03714 (14), 26.1141 (4)
β (°)	92.9914 (14)
*V* (Å^3^)	1284.03 (4)
*Z*	4
Radiation type	Cu *K*α
μ (mm^−1^)	3.84
Crystal size (mm)	0.15 × 0.12 × 0.02

Data collection	
Diffractometer	XtaLAB Synergy
Absorption correction	Multi-scan (*CrysAlis PRO*; Rigaku OD, 2023 ▸)
*T*_min_, *T*_max_	0.625, 1.000
No. of measured, independent and observed [*I* > 2σ(*I*)] reflections	49520, 2789, 2713
*R*_int_	0.038
(sin θ/λ)_max_ (Å^−1^)	0.639

Refinement	
*R*[*F* ^2^ > 2σ(*F* ^2^)], *wR*(*F* ^2^), *S*	0.031, 0.085, 1.05
No. of reflections	2789
No. of parameters	185
H-atom treatment	H atoms treated by a mixture of independent and constrained refinement
Δρ_max_, Δρ_min_ (e Å^−3^)	0.41, −0.38

Computer programs: *CrysAlis PRO* (Rigaku OD, 2023 ▸), *SHELXT* (Sheldrick, 2015*a* ▸), *SHELXL2019/3* (Sheldrick, 2015*b* ▸) and *XP* (Bruker, 1998 ▸).

## supplementary crystallographic information

### Crystal data

**Table:**

C_10_H_11_N_5_O_2_S_2_	*F*(000) = 616
*M**_r_* = 297.36	*D*_x_ = 1.538 Mg m^−^^3^
Monoclinic, *P*2_1_/*n*	Cu *K*α radiation, λ = 1.54184 Å
*a* = 5.44832 (9) Å	Cell parameters from 32085 reflections
*b* = 9.03714 (14) Å	θ = 3.4–79.7°
*c* = 26.1141 (4) Å	µ = 3.84 mm^−^^1^
β = 92.9914 (14)°	*T* = 100 K
*V* = 1284.03 (4) Å^3^	Plate, colourless
*Z* = 4	0.15 × 0.12 × 0.02 mm

### Data collection

**Table:**

XtaLAB Synergy diffractometer	2789 independent reflections
Radiation source: micro-focus sealed X-ray tube, PhotonJet (Cu) X-ray Source	2713 reflections with *I* > 2σ(*I*)
Mirror monochromator	*R*_int_ = 0.038
Detector resolution: 10.0000 pixels mm^-1^	θ_max_ = 80.3°, θ_min_ = 3.4°
ω scans	*h* = −6→6
Absorption correction: multi-scan (CrysAlisPro; Rigaku OD, 2023)	*k* = −11→11
*T*_min_ = 0.625, *T*_max_ = 1.000	*l* = −33→33
49520 measured reflections	

### Refinement

**Table:**

Refinement on *F*^2^	Primary atom site location: dual
Least-squares matrix: full	Hydrogen site location: mixed
*R*[*F*^2^ > 2σ(*F*^2^)] = 0.031	H atoms treated by a mixture of independent and constrained refinement
*wR*(*F*^2^) = 0.085	*w* = 1/[σ^2^(*F*_o_^2^) + (0.0457*P*)^2^ + 0.9076*P*] where *P* = (*F*_o_^2^ + 2*F*_c_^2^)/3
*S* = 1.05	(Δ/σ)_max_ = 0.001
2789 reflections	Δρ_max_ = 0.41 e Å^−^^3^
185 parameters	Δρ_min_ = −0.38 e Å^−^^3^
0 restraints	

### Special details

**Table:**

Geometry. All esds (except the esd in the dihedral angle between two l.s. planes) are estimated using the full covariance matrix. The cell esds are taken into account individually in the estimation of esds in distances, angles and torsion angles; correlations between esds in cell parameters are only used when they are defined by crystal symmetry. An approximate (isotropic) treatment of cell esds is used for estimating esds involving l.s. planes.

### Fractional atomic coordinates and isotropic or equivalent isotropic displacement parameters (Å^2^)

**Table:**

	*x*	*y*	*z*	*U*_iso_*/*U*_eq_	
N1	0.3855 (2)	0.19499 (14)	0.48875 (5)	0.0195 (3)	
H01	0.372 (4)	0.118 (3)	0.5077 (8)	0.032 (5)*	
C2	0.5437 (3)	0.19268 (16)	0.44955 (5)	0.0192 (3)	
N3	0.5680 (2)	0.31253 (13)	0.41996 (5)	0.0191 (2)	
C4	0.4203 (3)	0.42490 (16)	0.42942 (5)	0.0192 (3)	
N5	0.2456 (2)	0.43104 (13)	0.46351 (5)	0.0201 (3)	
C6	0.2256 (3)	0.31003 (16)	0.49263 (5)	0.0196 (3)	
N2	0.6629 (2)	0.06574 (14)	0.44327 (5)	0.0214 (3)	
N4	0.0509 (2)	0.30223 (15)	0.52541 (5)	0.0237 (3)	
H04A	−0.033 (4)	0.382 (3)	0.5295 (8)	0.032 (5)*	
H04B	0.050 (4)	0.231 (2)	0.5456 (8)	0.030 (5)*	
S1	0.44381 (7)	0.58924 (4)	0.39493 (2)	0.02499 (11)	
C7	0.7117 (3)	0.55651 (19)	0.35892 (7)	0.0323 (4)	
H7A	0.663476	0.504404	0.327051	0.048*	
H7B	0.829641	0.496006	0.379343	0.048*	
H7C	0.787821	0.651309	0.350761	0.048*	
S2	0.84801 (6)	0.05740 (4)	0.39640 (2)	0.02013 (11)	
O1	1.04319 (19)	0.16344 (13)	0.40001 (4)	0.0258 (2)	
O2	0.9209 (2)	−0.09690 (12)	0.39465 (4)	0.0290 (3)	
C8	0.6737 (3)	0.09070 (17)	0.33828 (6)	0.0226 (3)	
C9	0.4618 (3)	0.0090 (2)	0.32667 (7)	0.0312 (4)	
H9	0.404045	−0.061191	0.350305	0.037*	
C10	0.3369 (3)	0.0325 (2)	0.27967 (8)	0.0396 (4)	
H10	0.192098	−0.022469	0.270891	0.047*	
C11	0.4215 (4)	0.1354 (3)	0.24549 (7)	0.0416 (5)	
H11	0.334422	0.150368	0.213437	0.050*	
C12	0.6317 (4)	0.2166 (2)	0.25758 (7)	0.0361 (4)	
H12	0.688191	0.287312	0.233994	0.043*	
C13	0.7600 (3)	0.19439 (19)	0.30433 (6)	0.0272 (3)	
H13	0.904891	0.249408	0.312962	0.033*	

### Atomic displacement parameters (Å^2^)

**Table:**

	*U*^11^	*U*^22^	*U*^33^	*U*^12^	*U*^13^	*U*^23^
N1	0.0203 (6)	0.0178 (6)	0.0208 (6)	0.0047 (5)	0.0039 (5)	0.0021 (5)
C2	0.0166 (6)	0.0197 (7)	0.0211 (6)	0.0013 (5)	0.0003 (5)	−0.0005 (5)
N3	0.0187 (6)	0.0170 (6)	0.0218 (6)	0.0018 (4)	0.0018 (4)	−0.0003 (5)
C4	0.0188 (7)	0.0181 (7)	0.0204 (7)	−0.0002 (5)	−0.0008 (5)	−0.0014 (5)
N5	0.0206 (6)	0.0167 (6)	0.0231 (6)	0.0031 (4)	0.0028 (5)	0.0001 (5)
C6	0.0196 (7)	0.0182 (7)	0.0210 (6)	0.0029 (5)	0.0001 (5)	−0.0008 (5)
N2	0.0220 (6)	0.0191 (6)	0.0235 (6)	0.0047 (5)	0.0058 (5)	0.0019 (5)
N4	0.0247 (7)	0.0193 (6)	0.0278 (6)	0.0077 (5)	0.0081 (5)	0.0049 (5)
S1	0.0290 (2)	0.01943 (19)	0.0270 (2)	0.00336 (14)	0.00611 (15)	0.00326 (13)
C7	0.0398 (10)	0.0247 (8)	0.0337 (9)	−0.0005 (7)	0.0147 (7)	0.0017 (7)
S2	0.01881 (19)	0.01887 (18)	0.02305 (18)	0.00429 (12)	0.00438 (13)	0.00141 (13)
O1	0.0183 (5)	0.0310 (6)	0.0280 (5)	−0.0006 (4)	0.0017 (4)	−0.0004 (4)
O2	0.0339 (6)	0.0215 (6)	0.0327 (6)	0.0113 (5)	0.0126 (5)	0.0041 (4)
C8	0.0204 (7)	0.0241 (7)	0.0235 (7)	0.0046 (6)	0.0023 (5)	−0.0051 (6)
C9	0.0252 (8)	0.0308 (8)	0.0379 (9)	0.0001 (7)	0.0046 (6)	−0.0084 (7)
C10	0.0253 (8)	0.0486 (11)	0.0444 (10)	0.0002 (8)	−0.0028 (7)	−0.0176 (9)
C11	0.0375 (10)	0.0596 (13)	0.0271 (8)	0.0123 (9)	−0.0045 (7)	−0.0106 (8)
C12	0.0401 (10)	0.0437 (10)	0.0248 (8)	0.0089 (8)	0.0044 (7)	−0.0003 (7)
C13	0.0282 (8)	0.0290 (8)	0.0246 (7)	0.0035 (6)	0.0042 (6)	−0.0018 (6)

### Geometric parameters (Å, º)

**Table:**

N1—C6	1.3637 (18)	C9—C10	1.389 (3)
N1—C2	1.3725 (18)	C10—C11	1.385 (3)
C2—N2	1.3326 (18)	C11—C12	1.382 (3)
C2—N3	1.3410 (19)	C12—C13	1.389 (2)
N3—C4	1.3270 (18)	N1—H01	0.86 (2)
C4—N5	1.3379 (19)	N4—H04A	0.87 (2)
C4—S1	1.7451 (15)	N4—H04B	0.83 (2)
N5—C6	1.3398 (19)	C7—H7A	0.9800
C6—N4	1.3144 (19)	C7—H7B	0.9800
N2—S2	1.6279 (13)	C7—H7C	0.9800
S1—C7	1.8017 (17)	C9—H9	0.9500
S2—O1	1.4309 (12)	C10—H10	0.9500
S2—O2	1.4512 (11)	C11—H11	0.9500
S2—C8	1.7737 (16)	C12—H12	0.9500
C8—C13	1.389 (2)	C13—H13	0.9500
C8—C9	1.390 (2)		
			
C6—N1—C2	119.71 (13)	C12—C11—C10	120.59 (17)
N2—C2—N3	124.13 (13)	C11—C12—C13	119.80 (18)
N2—C2—N1	115.72 (13)	C8—C13—C12	119.11 (16)
N3—C2—N1	120.15 (13)	C6—N1—H01	119.8 (14)
C4—N3—C2	115.57 (12)	C2—N1—H01	119.8 (14)
N3—C4—N5	127.97 (13)	C6—N4—H04A	116.2 (14)
N3—C4—S1	119.57 (11)	C6—N4—H04B	118.6 (14)
N5—C4—S1	112.46 (10)	H04A—N4—H04B	123 (2)
C4—N5—C6	115.19 (12)	S1—C7—H7A	109.5
N4—C6—N5	119.77 (13)	S1—C7—H7B	109.5
N4—C6—N1	119.64 (13)	H7A—C7—H7B	109.5
N5—C6—N1	120.59 (13)	S1—C7—H7C	109.5
C2—N2—S2	117.22 (10)	H7A—C7—H7C	109.5
C4—S1—C7	102.30 (8)	H7B—C7—H7C	109.5
O1—S2—O2	116.23 (7)	C10—C9—H9	120.8
O1—S2—N2	114.00 (7)	C8—C9—H9	120.8
O2—S2—N2	104.44 (6)	C11—C10—H10	119.7
O1—S2—C8	107.80 (7)	C9—C10—H10	119.7
O2—S2—C8	105.75 (7)	C12—C11—H11	119.7
N2—S2—C8	108.09 (7)	C10—C11—H11	119.7
C13—C8—C9	121.62 (15)	C11—C12—H12	120.1
C13—C8—S2	118.46 (12)	C13—C12—H12	120.1
C9—C8—S2	119.86 (13)	C8—C13—H13	120.4
C10—C9—C8	118.34 (17)	C12—C13—H13	120.4
C11—C10—C9	120.54 (17)		
			
C6—N1—C2—N2	−168.87 (13)	C2—N2—S2—O2	−172.04 (11)
C6—N1—C2—N3	10.2 (2)	C2—N2—S2—C8	−59.77 (13)
N2—C2—N3—C4	174.90 (14)	O1—S2—C8—C13	7.00 (14)
N1—C2—N3—C4	−4.1 (2)	O2—S2—C8—C13	−117.95 (13)
C2—N3—C4—N5	−2.5 (2)	N2—S2—C8—C13	130.67 (12)
C2—N3—C4—S1	177.57 (10)	O1—S2—C8—C9	−175.82 (12)
N3—C4—N5—C6	2.6 (2)	O2—S2—C8—C9	59.23 (14)
S1—C4—N5—C6	−177.41 (11)	N2—S2—C8—C9	−52.15 (14)
C4—N5—C6—N4	−176.47 (13)	C13—C8—C9—C10	0.4 (2)
C4—N5—C6—N1	3.8 (2)	S2—C8—C9—C10	−176.69 (13)
C2—N1—C6—N4	170.22 (14)	C8—C9—C10—C11	−0.3 (3)
C2—N1—C6—N5	−10.1 (2)	C9—C10—C11—C12	−0.1 (3)
N3—C2—N2—S2	−0.3 (2)	C10—C11—C12—C13	0.3 (3)
N1—C2—N2—S2	178.74 (10)	C9—C8—C13—C12	−0.2 (2)
N3—C4—S1—C7	−6.77 (14)	S2—C8—C13—C12	176.96 (13)
N5—C4—S1—C7	173.26 (12)	C11—C12—C13—C8	−0.2 (3)
C2—N2—S2—O1	60.06 (13)		

### Hydrogen-bond geometry (Å, º)

**Table:**

*D*—H···*A*	*D*—H	H···*A*	*D*···*A*	*D*—H···*A*
N1—H01···N2^i^	0.86 (2)	2.11 (2)	2.9701 (18)	178 (2)
N4—H04*A*···N5^ii^	0.87 (2)	2.06 (2)	2.9245 (18)	177 (2)
N4—H04*B*···O2^i^	0.83 (2)	1.97 (2)	2.7912 (18)	166 (2)
C7—H7*C*···O2^iii^	0.98	2.63	3.445 (2)	140

Symmetry codes: (i) −*x*+1, −*y*, −*z*+1; (ii) −*x*, −*y*+1, −*z*+1; (iii) *x*, *y*+1, *z*.
